# Management of cT2N0M0 esophageal cancer: a systematic review and meta-analysis

**DOI:** 10.1093/dote/doag113

**Published:** 2026-10-20

**Authors:** Yousif Eliya, Samantha Leng, Virginia R Litle, Mohammad Asghari, Geoffrey P Kohn

**Affiliations:** Monash University, Department of Surgery, Melbourne, Victoria, Australia; Cabrini Health, Oesophagogastric Surgery Unit, Melbourne, Victoria, Australia; Eastern Health, Department of Surgery, Melbourne, Victoria, Australia; California Pacific Medical Center, Department of Thoracic and Cardiac Surgery, Sutter Health, San Francisco, CA, USA; Cabrini Health, Oesophagogastric Surgery Unit, Melbourne, Victoria, Australia; Monash University, Department of Surgery, Melbourne, Victoria, Australia; Cabrini Health, Oesophagogastric Surgery Unit, Melbourne, Victoria, Australia; Eastern Health, Department of Surgery, Melbourne, Victoria, Australia

**Keywords:** chemoradiotherapy, cT2N0 esophageal cancer, esophagectomy, multimodal therapy, neoadjuvant therapy

## Abstract

Management of cT2N0M0 esophageal cancer is challenging due to staging limitations and limited evidence. While neoadjuvant therapies benefit advanced disease, its role in cT2N0M0 remains unclear, compounded by underrepresentation in trials and the differing responses to treatment between adenocarcinoma (EAC) and squamous cell carcinoma (SCC). To address this, the ISDE conducted a systematic review and meta-analysis comparing outcomes of neoadjuvant therapy plus surgery (NeoAdj+S) versus primary surgical resection (PSR). MEDLINE, EMBASE, and CENTRAL were searched to 25 November 2024 for English-language studies of adults (≥18 years) with EAC or SCC staged as cT2N0M0. Case series with ≤7 patients, recurrent disease were excluded. Two independent reviewers (YE, SL) selected studies, and extracted data. A random-effects meta-analysis was performed to assess outcomes. Of 299 unique articles screened, 18 studies were included, totaling 14,566 cT2N0 patients. Pooled analysis revealed no statistically significant differences between NeoAdj+S and PSR in terms of 5-year overall survival (OS) (RR 1.08, 95% CI 0.85–1.36; favoring NeoAdj+S), 3-year OS (RR 1.10, 95% CI 0.92–1.30; favoring NeoAdj+S); 5-year disease-free survival (RR 0.88, 95% CI 0.70–1.12; favoring PSR), 30-day mortality (RR 0.91, 95% CI 0.53–1.54; favoring NeoAdj+S), overall complication rates (RR 0.97, 95% CI 0.87–1.08; favoring NeoAdj+S); surgical complications (RR 0.92, 95% CI 0.78–1.08; favoring NeoAdj+S); or medical complications (RR 0.86, 95% CI 0.61–1.23; favoring NeoAdj+S). All included studies demonstrated high risk-of-bias. No clear survival or perioperative advantage was found for NeoAdj+S over PSR. High-quality clinical trials are needed to guide treatment decisions.

## Graphical Abstract

**Graphical Abstract:**
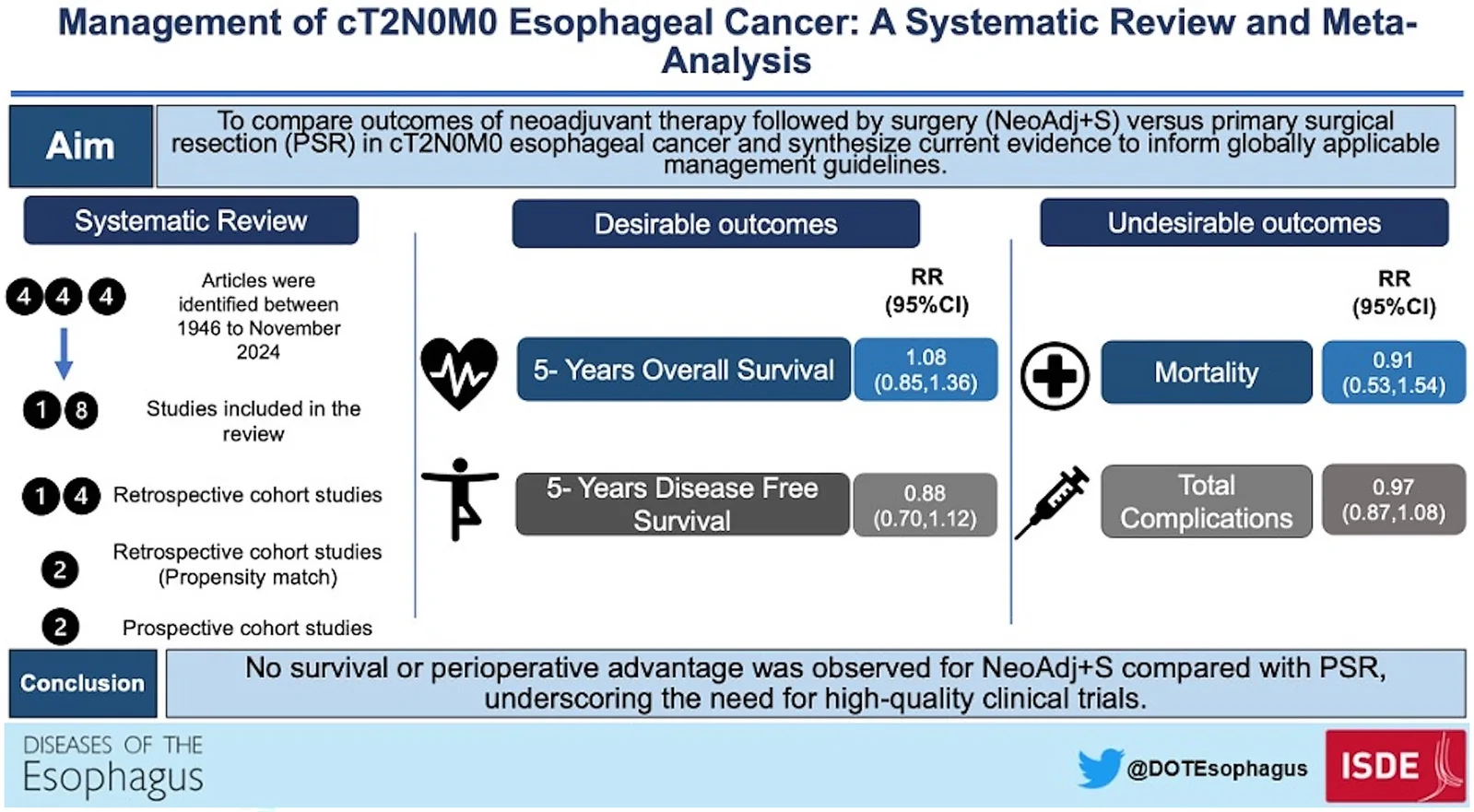

## INTRODUCTION/BACKGROUND

The incidence and mortality of esophageal cancer is rising,^1^ with an estimated 511,000 new cases and 445,000 deaths reported worldwide in 2022.^2^ The burden of esophageal cancer is global, with the majority of cases occurring in Asia (74.9%) followed by Europe (10.5%), Africa (5.9%), and Northern America (5.9%),^2^ Histologically, squamous cell carcinoma (SCC) remains the predominant pathological subtype in Eastern populations, whereas adenocarcinoma is increasingly common in developed Western Nations.^3^

Guidelines from the United States (US), Europe, and East Asia recommend local resection as the preferred treatment for early stage esophageal cancer.^4–6^ For advanced disease, guidelines support a more aggressive approach with multimodal therapy, typically involving neoadjuvant treatment.^4–6^ However, the optimal management of intermediate-stage esophageal cancer remains controversial. While European and US treatment algorithms support neoadjuvant treatment for patients with clinical T2N0M0 esophageal cancer, the Japanese Esophageal Society proposes there is some evidence for primary surgery in this patient group—this practice would eliminate neoadjuvant therapy related toxicity, shorten treatment duration and possibly reduce overtreatment and unnecessary healthcare burden.^4–6^ Further, clinicians treating intermediate stage esophageal cancers also face the challenge of inaccurate clinical staging. Despite the use of multimodal staging modalities— including endoscopic ultrasound (EUS), computerized tomography (CT), ultrasound (US), and positron emission tomography (PET)—accurate staging in T2N0 disease is achieved in as few as 19% of cases.^7^

To our knowledge, there are no randomized controlled trials that conclusively compare neoadjuvant therapy to primary surgery in a T2N0 cohort of patients with esophageal cancer. This lack of evidence underscores a significant gap in knowledge and presents a real-world challenge for clinicians managing this patient population. To address this, a systematic review and meta-analysis was conducted on behalf of the International Society for Disease of the Esophagus (ISDE), aiming to evaluate the current available evidence in this area. A secondary objective of this study is to inform the development of a globally accepted guideline for the management of clinical T2N0 esophageal cancer.

## MATERIAL AND METHODS

The conduct of this review followed the *Cochrane Handbook for Systematic Reviews of Interventions,*^8^ and reported findings using the Preferred Reporting Items for Systematic Reviews and Meta-Analyses (PRISMA) guidelines.^9^

### Eligibility criteria

Included studies met the following criteria: (1) adults (≥18 years) with cT2N0M0 esophageal cancer, including both adenocarcinoma (EAC) and squamous cell carcinoma (SCC); (2) tumors classified as Siewert type I, II, or III primary disease only; (3) patients undergoing chemotherapy, immunotherapy, chemoradiotherapy, or surgery; (4) studies published in English- language only. The exclusion criteria were (1) case series with ≤7 cT2N0 patients; (2) non-EAC/SCC histology; (3) abstract only studies; (4) patients with a history of other cancers (except non-melanoma skin cancers).

### Outcome measures

The desirable outcomes were: 5-year overall survival (OS) defined as time from treatment to death from any cause, 3-year OS, 5-year disease free survival (DFS) defined as time from treatment to disease recurrence, 3-year DFS, longest reported survival (months), longest reported DFS (months) and R0 resection rate. The undesirable outcomes included: 30-day perioperative mortality; overall complications; overall surgical complications (anastomotic leak, chylothorax, conduit necrosis, and recurrent laryngeal nerve injury); and overall medical complications (cardiovascular, pulmonary, infectious, and other complications).

### Search strategy

We searched the following databases:

- Ovid (MEDLINE—Epub, Ahead of print, in-progress & other indexed citations, MEDLINE daily)—searched from 1946 to 25 November 2024
- EMBASE—searched from 1947 to 25 November 2024
- Cochrane Central Register of Controlled Trials (CENTRAL)—searched on 25 November 2024
- ClincalTrials.gov on 25 November 2024

We searched using a combination of medical subject heading (MeSH), title, and abstract terms encompassing ‘Oesophageal Cancer’; and ‘cT2N0M0’ following the guidelines of an experienced research librarian (DH). We further modified search terms to the EMBASE database to better fit the database mapping index. A full list of included terms is available in Table 1 of the Supplementary Appendix.

We also searched ClinicalTrials.org (https://clinicaltrials.gov), Australian New Zealand Clinical Trials Registry (https://www.anzctr.org.au/) and the WHO ICTRP portal (http://www.who.int/ictrp/en) on 19 November 2024 for ongoing clinical trials or unpublished results.

### Selection of studies

Two reviewers (YE and SL) independently screened titles, abstracts, and full texts. Any discrepancies were carried forward to the full-text stage and resolved by a senior reviewer (GK). Reasons for study exclusion were documented.

### Data extraction and management

Data were collected and recorded into predetermined tables independently by the reviewers in Covidence™. The following information was sought and extracted from each study, where available:

- Baseline characteristics: primary author and year, country, mean age (years), male sex (%), tumor location (upper, middle, end-third), histology (EAC, SCC, other), chemotherapy or chemoradiotherapy protocol, and staging modality.
- Neoadjuvant case definitions: chemoradiotherapy, chemotherapy alone, radiotherapy alone, or a combination of these treatments.
- Outcomes: OS, longest reported survival, DFS, longest reported DFS, perioperative mortality, R0 resection rate, overall complications, surgical complications, and medical complications. All data extracted were presented separately.

### Assessment of risk of bias in included studies

Risk of bias for observational cohort studies was assessed using the Newcastle–Ottawa Scale (NOS) for cohort studies.^10^ The NOS evaluates three domains: Selection (4 items, 4 points), Comparability (1 item, 2 points), and Outcome (3 items, 3 points). Two reviewers (YE, SL) independently applied the NOS criteria to each study and assigned scores according to the published guidelines. Discrepancies were resolved through discussion, with a third reviewer (GK) consulted if needed. Total NOS scores (0–9 points) were used to classify studies as low (7–9 points), moderate (4–6 points), or high (0–3 points) risk of bias.

### Data synthesis and analysis

For categorical outcomes, proportions with 95% confidence intervals were extracted; for continuous outcomes, means and standard deviations were used, with medians and ranges converted when necessary. Random-effects meta-analyses were performed using the restricted maximum likelihood (REML) method^9^^,^^11^^,^^12^ to account for between-study heterogeneity, which may arise from unpublished studies, geographical differences, and other unknown sources. A continuity correction of 0.5 was applied to studies with zero cells. Pooled estimates were expressed as odds or risk ratios with 95% confidence intervals for categorical outcomes. Heterogeneity was assessed using Cochran’s Q test, τ,^2^ and I^2^ statistics, with I^2^ values below 75% considered acceptable.^12^ Publication bias was evaluated through funnel plots and tested using Egger’s and Begg’s methods,^11^^,^^13^ with the trim-and-fill approach applied when bias was detected.^11^ Meta-regression analyses explored potential moderators (e.g. mean age, male sex, and study sample size), and subgroup analyses were conducted based on study design, chemotherapy protocol, chemoradiotherapy protocol, and neoadjuvant case definitions. Sensitivity analyses using a leave-one-out approach assessed robustness. All statistical analyses were conducted by a senior statistician (MA) using Stata version 18.5 (StataCorp, College Station, TX, USA).

## RESULTS

### Literature search

The search yielded 444 citations, of which 18 articles were included in the review (Fig. 1). After duplicate screening, 136 articles were excluded at full-text stages, with detailed reasoning reported in Figure 1.

**Fig. 1:**
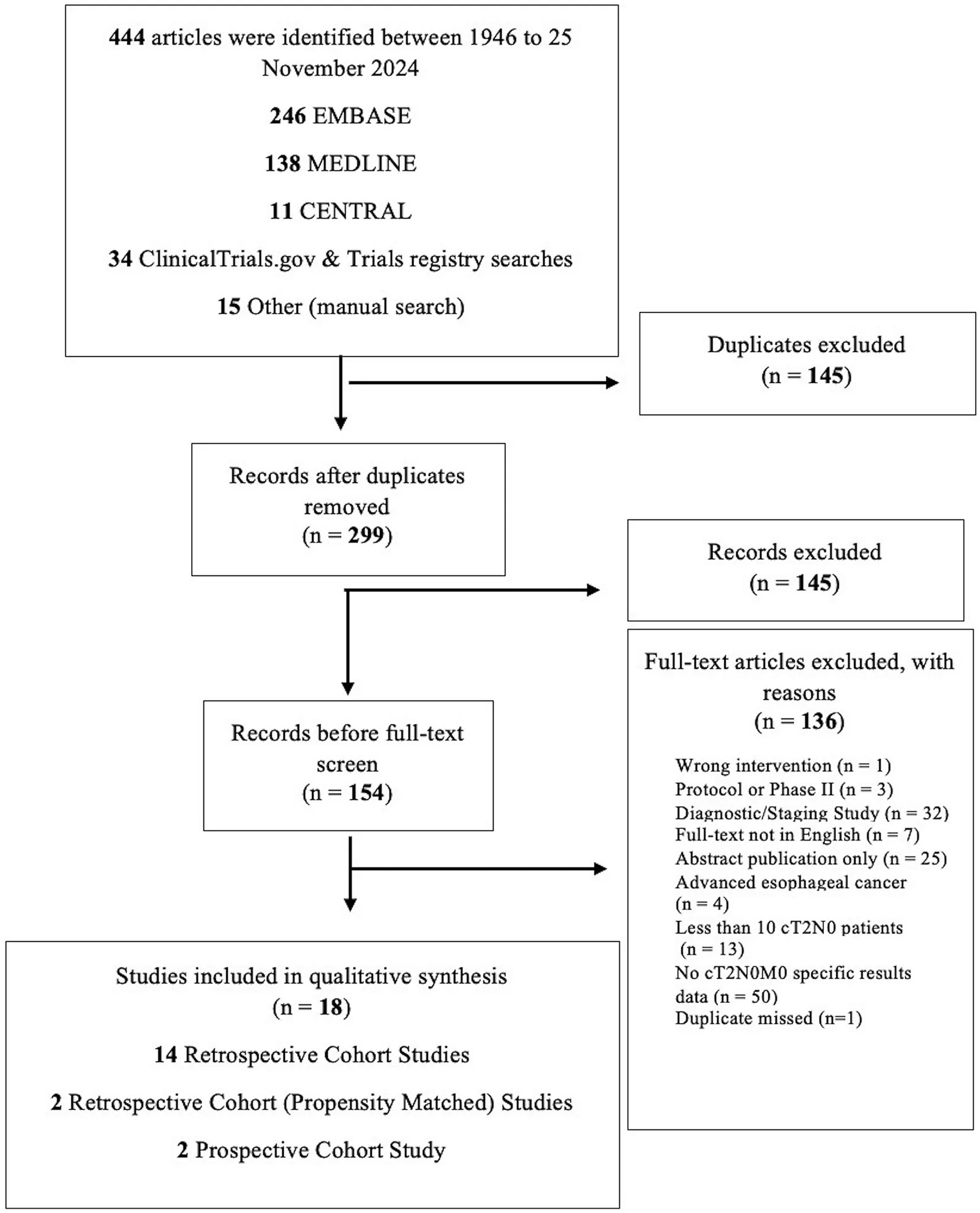
PRIMSA chart of the included studies.

### Study characteristics

The 18 included studies represented 14,566 patients (7936 NeoAdj+S versus 6630 PSR) with mean age of 63.7 years (SD 2.5). Majority (11 of 18 studies) were conducted in the USA,^14–24^ four in Europe,^25–27^ and three in China/Taiwan^28–30^ (Table 1).

**Table 1:** Study characteristics

**Study**	**Country**	**Study Design**	**Neoadjuvant Therapy Type**	**Total Sample (NeoAdj + S/PSR)**	**Male (%)**	**Mean age**	**EAS**	**SCC**
Capovilla, 2021	Italy	Retrospective cohort	CRT	229 (38/191)	83.8	61.2	96	133
Chen, 2012	Taiwan (China)	Retrospective cohort	CRT	71 (57/14)	90.2	57.4	–	71
Crabtree, 2013	USA	Retrospective cohort	CRT + CT + RT	752 (270/482)	–	63.8	182	29
Dolan, 2016	USA	Retrospective cohort	CRT	27 (11/16)	93.4	65.9	25	2
Goense, 2018	Netherlands	Retrospective cohort	CRT	533 (535/180)	74.4	64.3	422	111
Hardacker, 2014	USA	Prospective cohort	CRT	68 (33/35)	80.9	61.7	57	11
Lin, 2024	China	Retrospective cohort	CRT	583(316/267)	78.6	–	423	160
Malin, 2009	USA	Prospective cohort	CRT	216 (88/128)	–	–	–	33
Markar, 2016	France	Retrospective cohort	CRT + CT	355 (70/285)	80.6	–	171	184
Martin, 2013	USA	Retrospective cohort	RT	490 (223/267)	77.6	64.9	–	–
PerezHolguin, 2021	USA	Retrospective cohort	CRT	1300 (821/479)	–	–	–	–
Rhodin, 2021	USA	Retrospective cohort	CRT + CT	2540 (1363/1177)	82.8	64.4	–	461
Rodriguez-Quintero, 2024	USA	Retrospective cohort	CRT	3413(2359/1054)	88.1	64.7	3413	–
Samson, 2016	USA	Retrospective cohort	CRT + CT	1785 (853/932)	82.7	65.5	–	–
Song, 2016	China	Retrospective cohort	RT	243(151/92)	–	–	–	–
Speicher, 2014	USA	Retrospective cohort	CRT + CT + RT	1559 (688/871)	84.0	63.8	–	–
Wirsik^a^, 2024	Germany	Retrospective cohort	CRT + CT	333 (187/146)	82.3	67.3	–	–
Zhang, 2012	USA	Retrospective cohort	CRT	69 (55/14)	85.5	62.6	54	15

Abbreviations: Chemoradiotherapy (CRT); Chemotherapy (CT); Radiotherapy (RT); Neoadjuvant therapy followed by surgery (NeoAdj+S); primary surgical resection (PSR); Esophageal Adenosarcoma (EAS); Squamous cell carcinoma (SCC).

a This study used FLOT and CROSS protocols.

**Table 2:** Neoadjuvant therapy characteristics

**Study**	**Histology**	**Neoadjuvant Therapy Type**	**Chemotherapy Regimen**	**Radiotherapy Dose (Gy)**
Capovilla, 2021	EAC/SCC	CRT	Cisplatin +5-FU	–
Chen, 2012	SCC	CRT	Platinum +5-FU/RT	30 Gy
Crabtree, 2013	EAC/SCC/Other	CRT + CT + RT	Not reported	–
Dolan, 2016	EAC/SCC/Other	CRT	Platinum+5-FU; or capecitabine; or taxane-based + platinum	50.4–54 Gy
Goense, 2018	EAC/SCC	CRT	Carboplatin + Paclitaxel (CROSS regimen)	41.4 Gy
Hardacker, 2014	EAC/SCC	CRT	Not reported	–
Lin, 2024	EAC/SCC	CRT	Not reported	–
Malin, 2009	SCC	CRT	Platinum +5-FU/RT	45–50 Gy
Markar, 2016	EAC/SCC	CRT + CT	Not reported	–
Martin, 2013	Mix	RT	RT	–
PerezHolguin, 2021	Mix	CRT	Not reported	–
Rhodin, 2021	SCC	CRT + CT	Not reported	–
Rodriguez-Quintero, 2024	EAC	CRT	Not reported	40–45 Gy
Samson, 2016	Mix	CRT + CT	Not reported	–
Song, 2016	Mix	RT	RT	–
Speicher, 2014	Mix	CRT + CT + RT	Not reported	–
Wirsik^a^, 2024	Mix	CRT + CT	Not reported^a^	–
Zhang,2012	EAC/SCC	CRT	Platinum +5-FU/RT	<44 Gy or > 44 Gy (unknown in some patients)

Abbreviations: Chemoradiotherapy (CRT); Chemotherapy (CT); Radiotherapy (RT); Esophageal Adenosarcoma (EAC); Squamous cell carcinoma (SCC).

a This study used FLOT and CROSS protocols.

Among the included studies, 10 studies were neoadjuvant chemoradiotherapy, four mixed neoadjuvant therapy (chemoradiotherapy + chemotherapy), two mixed neoadjuvant therapy (chemoradiotherapy + chemotherapy + radiotherapy) and two neoadjuvant radiotherapy vs. PSR. Three studies specified inclusion criteria for Siewart classification, including only Siewart I and II participants.^26^^,^^27^^,^^31^ Siewart classification was not stated in the other 15 studies. Characteristics of the neoadjuvant therapy protocols are described in Table 2.

### 5-year overall survival

Eleven studies (nine retrospective and two prospective cohort) reported 5-year OS.^16–20^^,^^23–25^^,^^27^^,^^29^^,^^31^

Overall, NeoAdj+S was associated with a non-significant 8% increase in 5-year OS compared to PSR (RR 1.08, 95% CI 0.85–1.36; I^2^ = 93.8%; high heterogeneity, favors NeoAdj+S) (Fig. 2). Meta-regression identified no significant predictors of 5-year OS.

**Fig. 2:**
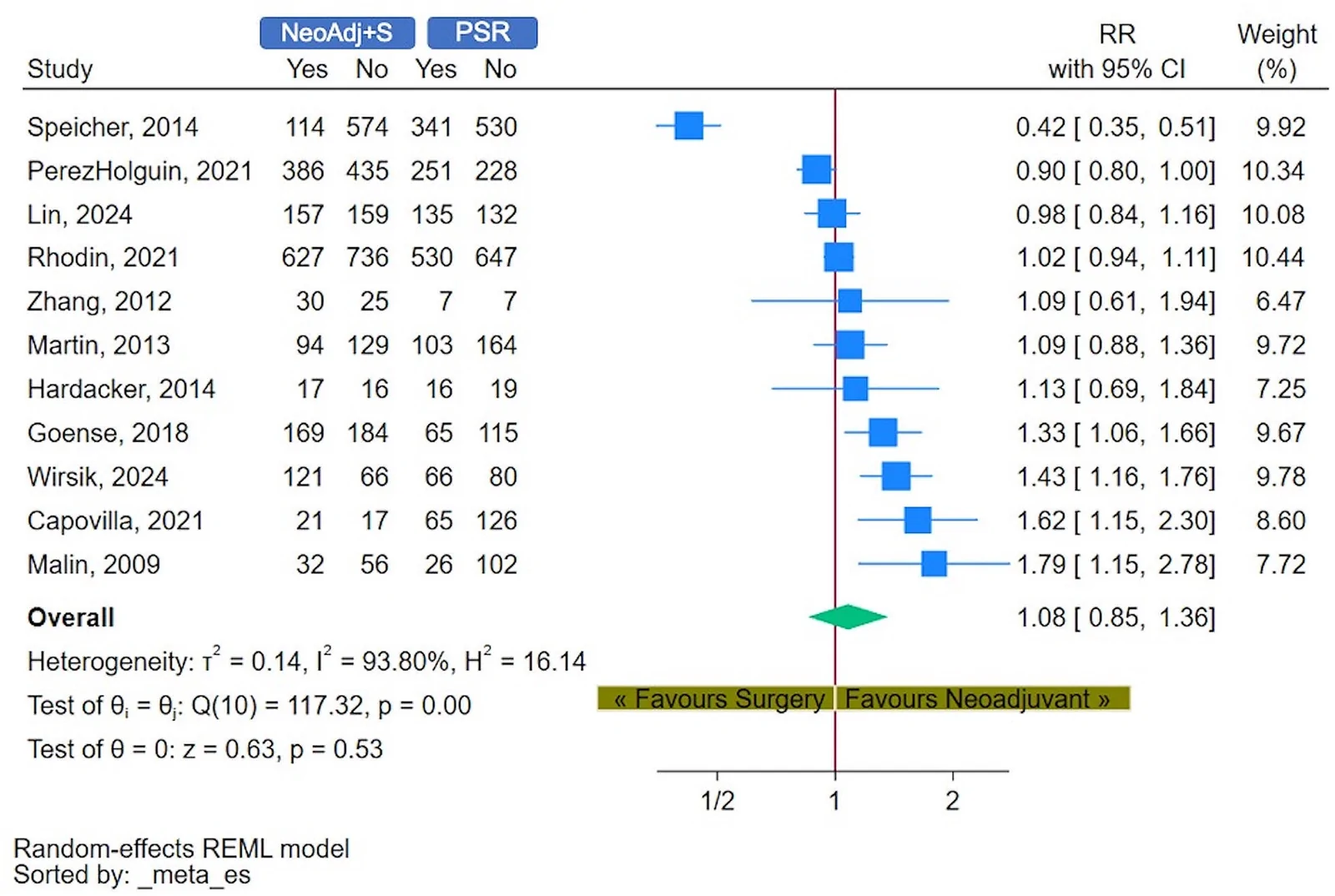
Forest plot of 5-year Overall Survival between Neoadjuvant therapy followed by surgery (NeoAdj + S) and Primary surgical resection (PSR). RR, risk ratios; IV, inverse variance; df, degrees of freedom.

Across neoadjuvant therapy subgroups, chemoradiotherapy was associated with a non-significant 19% increase in 5-year OS compared to PSR (RR 1.19, 95% CI 0.97–1.45; I^2^ = 76.4%; moderate heterogeneity, favors NeoAdj+S) (Fig. 1A). In one study,^23^ mixed chemoradiotherapy + chemotherapy+ radiotherapy was associated with a significant 58% reduction in 5-year OS compared to PSR (RR 0.42, 95% CI 0.35–0.51).

Across chemoradiotherapy protocols subgroups, the CROSS regimen was reported in two studies and demonstrated a 38% higher, significant, 5-year OS (RR 1.38, 95% CI 1.19–1.61; I^2^ = 0%, low heterogeneity) (Fig. 1B supplementary). The FLOT regimen (fluorouracil, leucovorin, oxaliplatin, and docetaxel) was reported in one study and demonstrated a 43% higher, significant, 5-year OS (RR 1.43, 95% CI 1.16–1.76; I^2^ = 0%, low heterogeneity) (Fig. 1C supplementary). Most studies did not specify the chemoradiotherapy protocol used.

Across study design subgroups, retrospective cohort was reported in nine studies and demonstrated a 3% higher, but non-significant, 5-year OS in NeoAdj+S compared to PSR (RR 1.03, 95% CI 0.79–1.33; I^2^ = 95.0%) (Fig. 1D supplementary).

### 3-year overall survival

Four studies (three retrospective and one prospective cohort) reported 3-year OS.^17^^,^^21^^,^^25^^,^^27^

Overall, NeoAdj+S was associated with a non-significant 10% increase in 3-year OS compared to PSR (RR 1.10, 95% CI 0.92–1.30; I^2^ = 81.0%; high heterogeneity, favors NeoAdj+S) (Fig. 3).

**Fig. 3:**
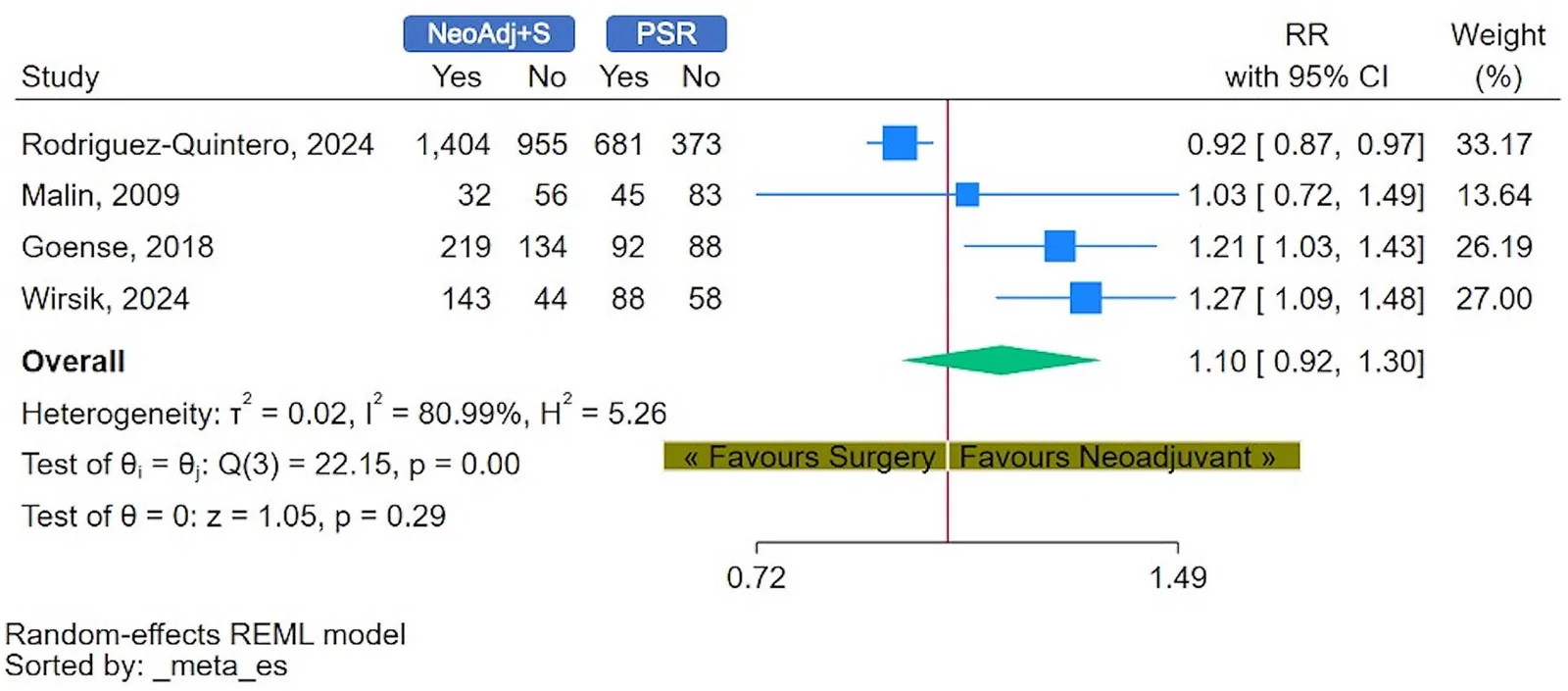
Forest plot of 3-year Overall Survival between Neoadjuvant therapy followed by surgery (NeoAdj + S) and Primary surgical resection (PSR). RR, risk ratios; IV, inverse variance; df, degrees of freedom.

Across neoadjuvant therapy subgroups, chemoradiotherapy was associated with a non-significant 4% increase in 3-year OS compared to PSR (RR 1.04, 95% CI 0.85–1.26; I^2^ = 75.9%; substantial heterogeneity, favors NeoAdj+S) (Fig. 2A). In one study,^27^ mixed chemoradiotherapy + chemotherapy+ radiotherapy was associated with a significant 27% increase in 3-year OS compared to PSR (RR 1.27, 95% CI 1.09–1.48).

Across chemoradiotherapy protocols subgroups, the CROSS regimen was reported in two studies and demonstrated a 24% higher, significant, 3-year OS (RR 1.24, 95% CI 1.11–1.39; I^2^ = 0%, low heterogeneity) (Fig. 2B supplementary), the FLOT regimen was reported in one study and demonstrated a 27% higher, significant, 3-year OS (RR 1.27, 95% CI 1.09–1.48; I^2^ = 0%, low heterogeneity) (Fig. 2C supplementary). Most studies did not specify the chemoradiotherapy protocol used.

Across study design subgroups, retrospective cohort was reported in three studies and demonstrated an 11% higher, but non-significant, 3-year OS in NeoAdj+S compared to PSR (RR 1.11, 95% CI 0.90–1.37; I^2^ = 88.3%, high heterogeneity) (Fig. 2D supplementary).

### Disease-free survival

Four studies (all retrospective) reported 5-year DFS.^18^^,^^24^^,^^28^^,^^29^

Overall, NeoAdj+S was associated with a non-significant 12% decrease in 5-year DFS compared to PSR (RR 0.88, 95% CI 0.70–1.12; I^2^ = 76.4%; moderate heterogeneity, favors PSR) (Fig. 4).

**Fig. 4:**
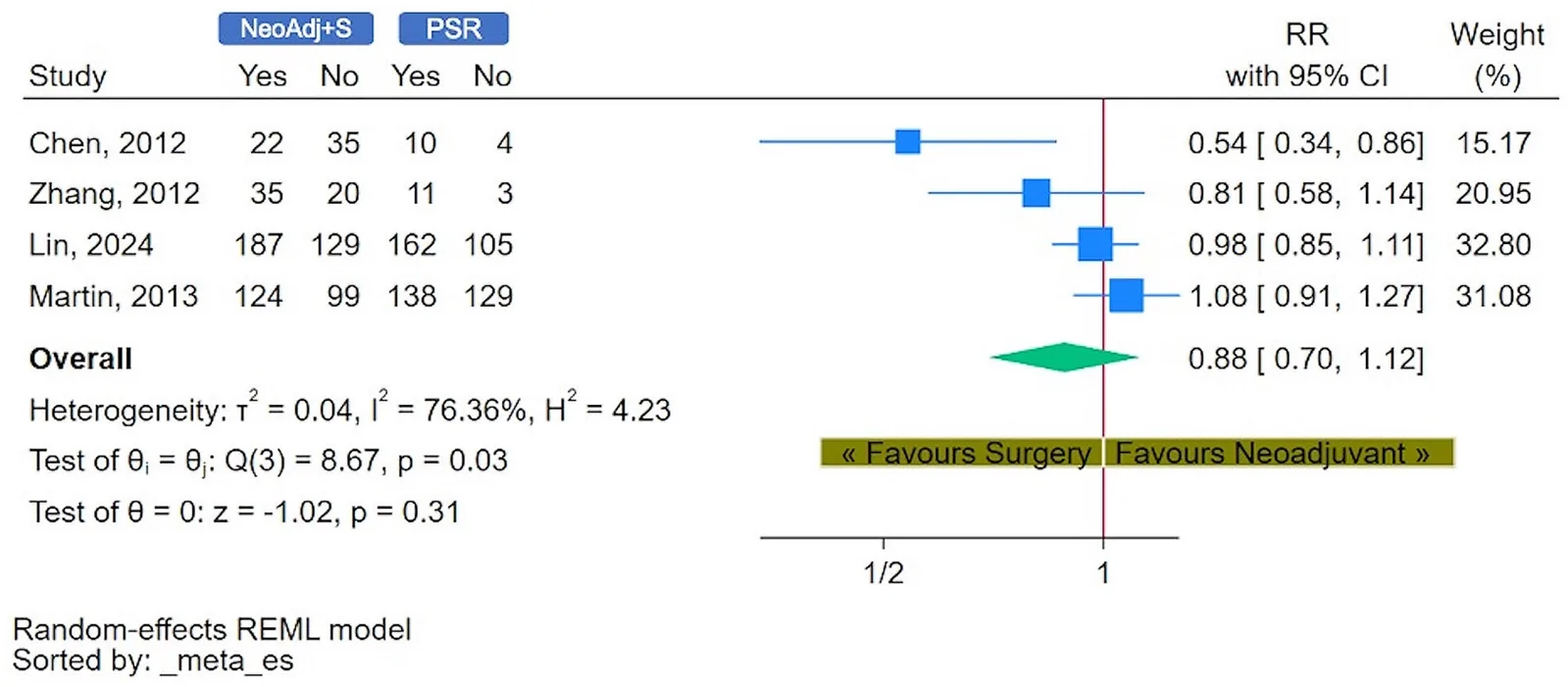
Forest plot of 5-year Disease-Free Survival between Neoadjuvant therapy followed by surgery (NeoAdj + S) and Primary surgical resection (PSR). RR, risk ratios; IV, inverse variance; df, degrees of freedom.

Across neoadjuvant therapy subgroups, chemoradiotherapy was associated with a non-significant 20% decrease in 5-year DFS compared to PSR (RR 0.80, 95% CI 0.58–1.10; I^2^ = 69.7%; moderate heterogeneity, favors PSR) (Fig. 5). In one study,^17^ radiotherapy was associated with a non-significant 8% increase in 5-year DSR compared to PSR (RR 1.08, 95% CI 0.91–1.27).

**Fig. 5:**
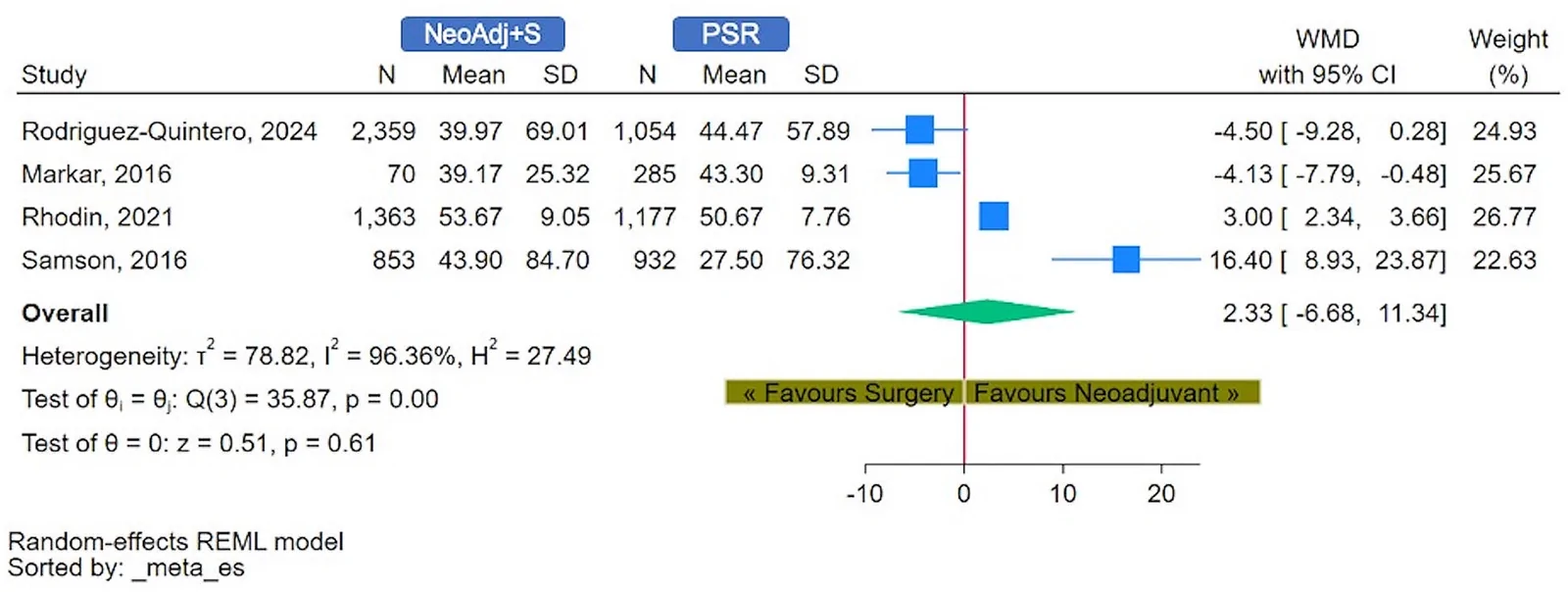
Forest plot of longest reported survival between Neoadjuvant therapy followed by surgery (NeoAdj + S) and Primary surgical resection (PSR). RR, risk ratios; IV, inverse variance; df, degrees of freedom.

Across chemoradiotherapy and chemotherapy only subgroups, most studies did not specify the treatment protocol used.

Across study design subgroups, retrospective cohort was reported in five studies and demonstrated a 12% lower, but non-significant, 5-year DFS in NeoAdj+S compared to PSR (RR 0.88, 95% CI 0.70–1.12; I^2^ = 78.4%, substantial heterogeneity) (Fig. 5A supplementary).

### Longest reported survival

Four studies (all retrospective) reported longest reported survival.^20–22^^,^^26^

Overall, NeoAdj+S was associated with a non-significant increase in longest reported survival compared to PSR (WMD (weighted mean difference) 2.33, 95% CI –6.68–11.34; I^2^ = 96.4%; high heterogeneity, favors NeoAdj+S) (Fig. 5).

Across neoadjuvant therapy subgroups, chemoradiotherapy was associated with a non-significant decrease in longest reported survival compared to PSR (WMD –4.50, 95% CI –9.28–0.28; I^2^ = 0%; low heterogeneity, favors PSR) (Fig. 4A supplementary).

Across chemoradiotherapy and chemotherapy only subgroups, most studies did not specify the treatment protocol used.

Across study design subgroups, retrospective cohort was reported in four studies and demonstrated a non-significant longest reported survival in NeoAdj+S compared to PSR (WMD 2.33, 95% CI –6.68–11.34; I^2^ = 96.4%; high heterogeneity, favors NeoAdj+S) (Fig. 4D supplementary).

### R0 resection rate

Seven studies (all retrospective) reported R0 resection rate.^15^^,^^24–28^^,^^31^ Meta-regression identified no significant predictors of R0 resection rate.

Overall, NeoAdj+S was associated with a non-significant in R0 resection rate compared to PSR (RR 1.00, 95% CI 0.89–1.12; I^2^ = 91.7%, high heterogeneity) (Fig. 6).

**Fig. 6:**
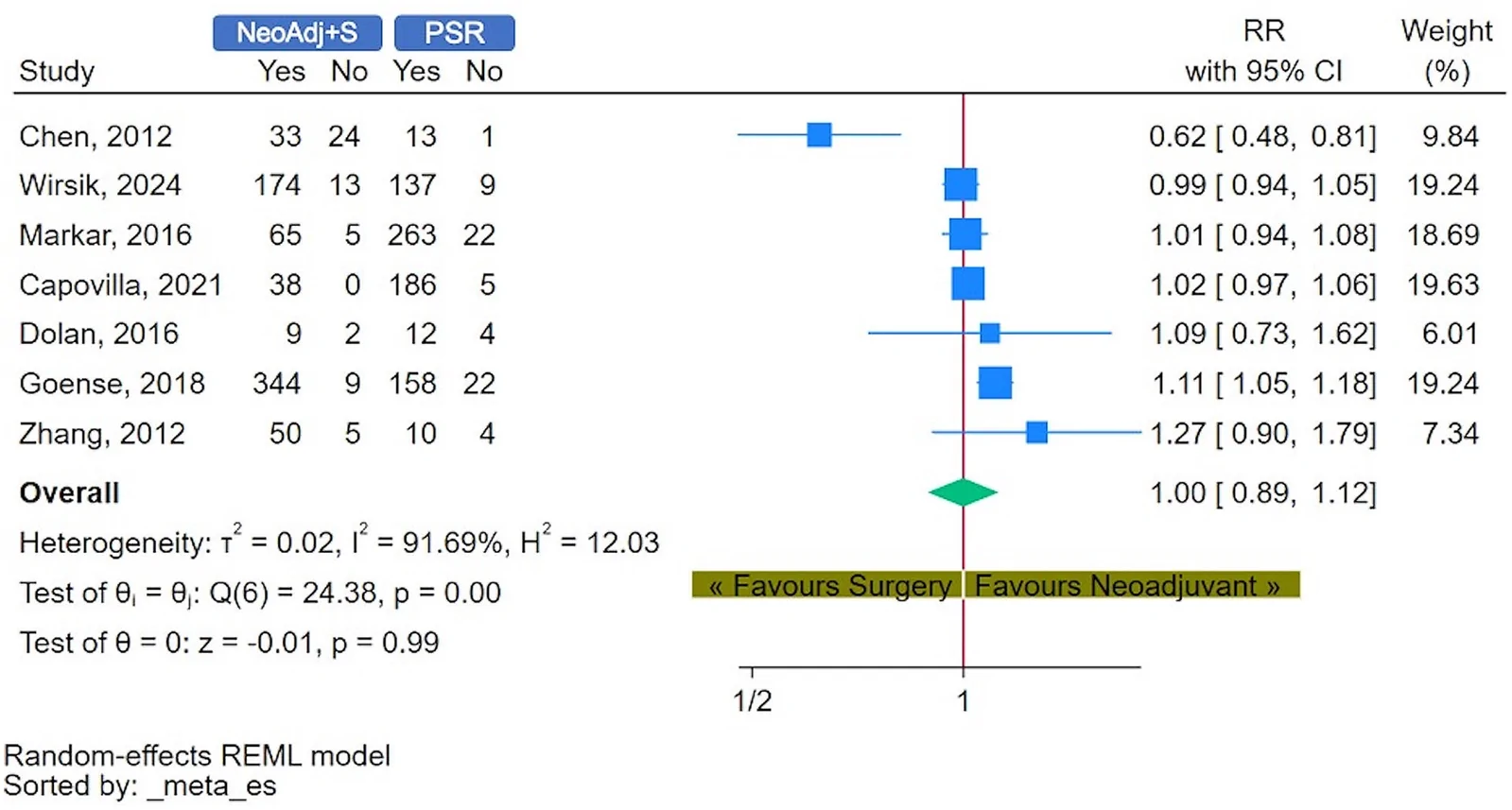
Forest plot of R0 resection rate between Neoadjuvant therapy followed by surgery (NeoAdj + S) and Primary surgical resection (PSR). RR, risk ratios; IV, inverse variance; df, degrees of freedom.

Across neoadjuvant therapy subgroups, chemoradiotherapy was associated with a non-significant 1% decrease in R0 resection rate compared to PSR (RR 0.99, 95% CI 0.79–1.24; I^2^ = 95.5%, high heterogeneity) (Fig. 5A supplementary).

Across chemoradiotherapy protocols subgroups, the CROSS regimen was reported in two studies and demonstrated a 5% higher, significant, R0 resection rate (RR 1.05, 95% CI 0.94–1.17; I^2^ = 86.7%, high heterogeneity) (Fig. 5B supplementary), while most studies did not specify the chemoradiotherapy protocol used.

Across chemotherapy protocols subgroups, the FLOT regimen was reported in one study and demonstrated a 0.01% lower, non-significant, R0 resection rate (RR 0.99, 95% CI 0.94–1.05) (Fig. 5C supplementary), while most studies did not specify the chemotherapy protocol used.

Across study design subgroups, retrospective cohort was reported in seven studies and demonstrated a non-significant R0 resection rate in NeoAdj+S compared to PSR (RR 1.00, 95% CI 0.89–1.12; I^2^ = 91.7%) (Fig. 5D supplementary).

### Perioperative mortality

Five studies (all retrospective) reported 30-day perioperative mortality.^14^^,^^23^^,^^25^^,^^26^^,^^28^

Overall, NeoAdj+S was associated with a non-significant 9% decrease in perioperative mortality compared to PSR (RR 0.91, 95% CI 0.53–1.54; I^2^ = 47.8%; moderate heterogeneity, favors NeoAdj+S) (Fig. 7).

**Fig. 7:**
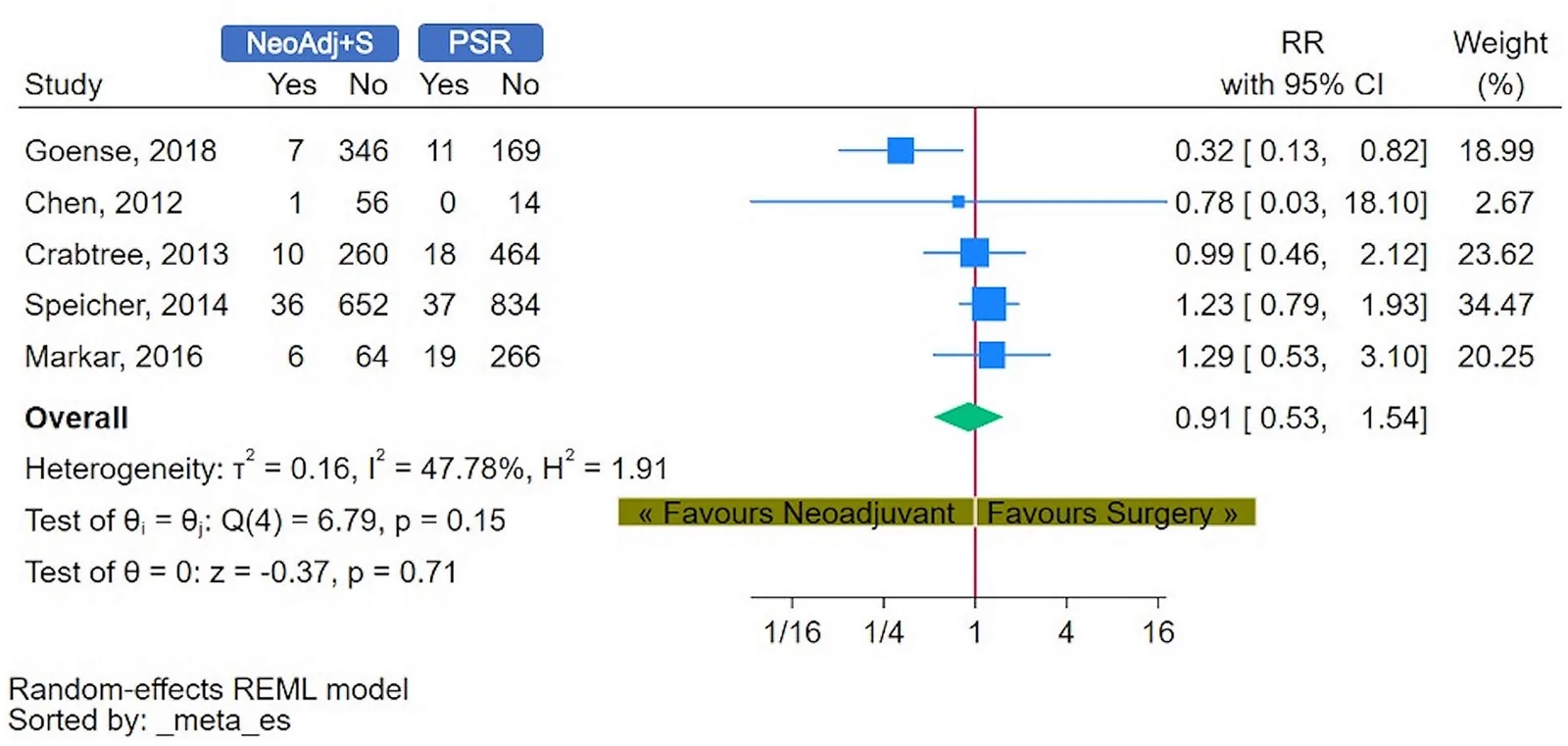
Forest plot of 30-day perioperative mortality between Neoadjuvant therapy followed by surgery (NeoAdj + S) and Primary surgical resection (PSR). RR, risk ratios; IV, inverse variance; df, degrees of freedom.

Across neoadjuvant therapy subgroups, chemoradiotherapy was associated with a significant 65% decrease in perioperative mortality compared to PSR (RR 0.35, 95% CI 0.14–0.85; I^2^ = 0%; low heterogeneity, favors NeoAdj+S) (Fig. 6A).

Across chemoradiotherapy protocols subgroups, the CROSS regimen was reported in one study and demonstrated a 68% decrease, significant, perioperative mortality (RR 0.32, 95% CI 0.13–0.82; I^2^ = 0%, low heterogeneity) (Fig. 6B supplementary), while most studies did not specify the chemoradiotherapy protocol used.

Across chemotherapy protocols subgroups, most studies did not specify the chemotherapy protocol used.

Across study design subgroups, retrospective cohort was reported in five studies and demonstrated a 9% decrease, but non-significant, perioperative mortality in NeoAdj+S compared to PSR (RR 0.91, 95% CI 0.53–1.54; I^2^ = 47.8%, moderate heterogeneity) (Fig. 6D supplementary).

### Overall complications

Five studies (four retrospective, one prospective) reported overall complications.^14^^,^^17^^,^^24^^,^^26^^,^^31^

Overall, NeoAdj+S was associated with a non-significant 3% decrease in overall complications compared to PSR (RR 0.97, 95% CI 0.87–1.08; I^2^ = 0%; low heterogeneity, favors NeoAdj+S) (Fig. 8).

**Fig. 8:**
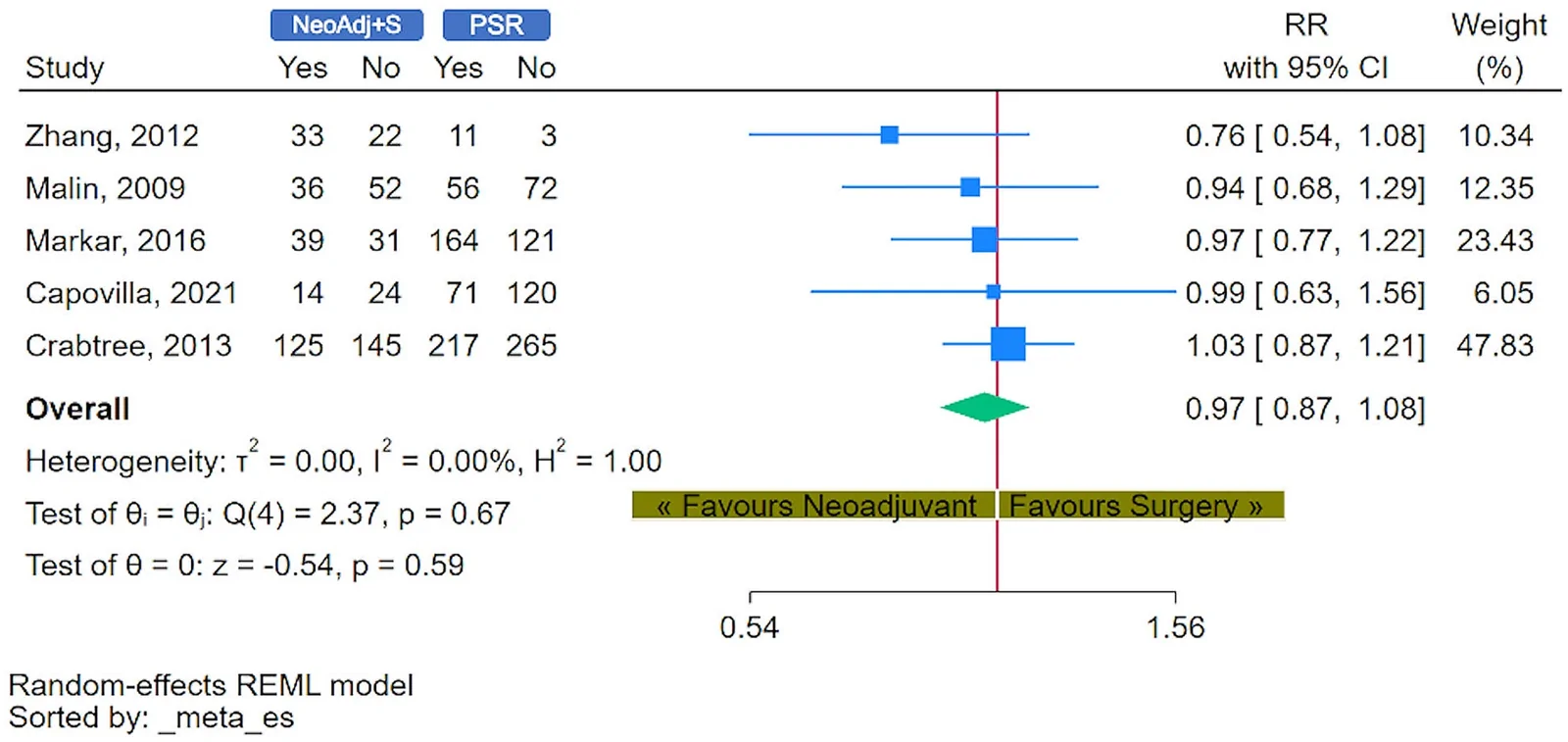
Forest plot of overall complications between Neoadjuvant therapy followed by surgery (NeoAdj + S) and Primary surgical resection (PSR). RR, risk ratios; IV, inverse variance; df, degrees of freedom.

Across neoadjuvant therapy subgroups, chemoradiotherapy was associated with a non-significant 12% decrease in overall complications compared to PSR (RR 0.88, 95% CI 0.71–1.08; I^2^ = 0%; low heterogeneity, favors NeoAdj+S) (Fig. 7A).

Across chemoradiotherapy protocols subgroups, most studies did not specify the chemoradiotherapy protocol used.

Across chemotherapy protocols subgroups, most studies did not specify the chemotherapy protocol used.

Across study design subgroups, retrospective cohort was reported in four studies and demonstrated a 3% decrease, but non-significant, overall complications in NeoAdj+S compared to PSR (RR 0.97, 95% CI 0.86–1.10; I^2^ = 0%, low heterogeneity) (Fig. 7D supplementary).

### Surgical complications

Five studies (four retrospective, one prospective) reported surgical complications.^14^^,^^17^^,^^24^^,^^26^^,^^31^

Overall, NeoAdj+S was associated with a non-significant 8% decrease in surgical complications compared to PSR (RR 0.92, 95% CI 0.78–1.08; I^2^ = 0%; low heterogeneity, favors NeoAdj+S) (Fig. 9).

**Fig. 9:**
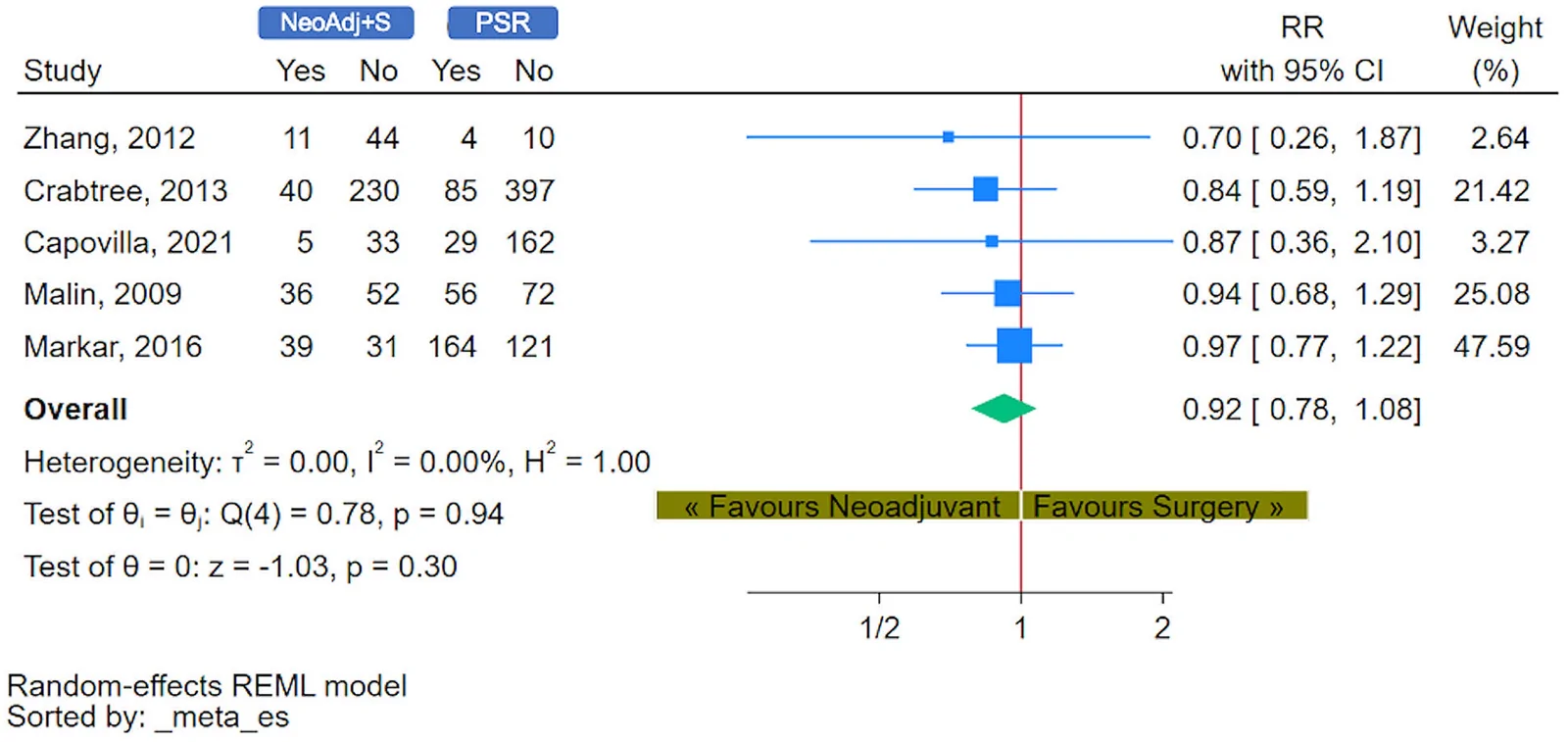
Forest plot of surgical complications between Neoadjuvant therapy followed by surgery (NeoAdj + S) and Primary surgical resection (PSR). RR, risk ratios; IV, inverse variance; df, degrees of freedom.

Across surgical complications, anastomotic leak was associated with a non-significant 12% decrease in NeoAdj+S compared to PSR (RR 0.88, 95% CI 0.56–1.36; I^2^ = 46.6%; moderate heterogeneity, favors NeoAdj+S) (Fig. 8A supplementary). NeoAdj+S was associated with a non-significant 92% increase in chylothorax complication compared to PSR (RR 1.92, 95% CI 0.90–4.07; I^2^ = 14.8%; low heterogeneity, favors PSR) (Fig. 8B supplementary). NeoAdj+S was associated with a non-significant 92% increase in conduit necrosis compared to PSR (RR 1.92, 95% CI 0.90–4.07; I^2^ = 14.8%; low heterogeneity, favors PSR) (Fig. 8C supplementary). NeoAdj+S was associated with a non-significant 51% increase in laryngeal nerve injury compared to PSR (RR 2.51, 95% CI 0.80–7.93; favors PSR) (Fig. 8D supplementary).

### Medical complications

Three studies (three retrospective) reported medical complications.^14^^,^^24^^,^^31^

Overall, NeoAdj+S was associated with a non-significant 14% decrease in medical complications compared to PSR (RR 0.86, 95% CI 0.61–1.23; I^2^ = 86.2%; high heterogeneity, favors NeoAdj+S) (Fig. 10).

**Fig. 10:**
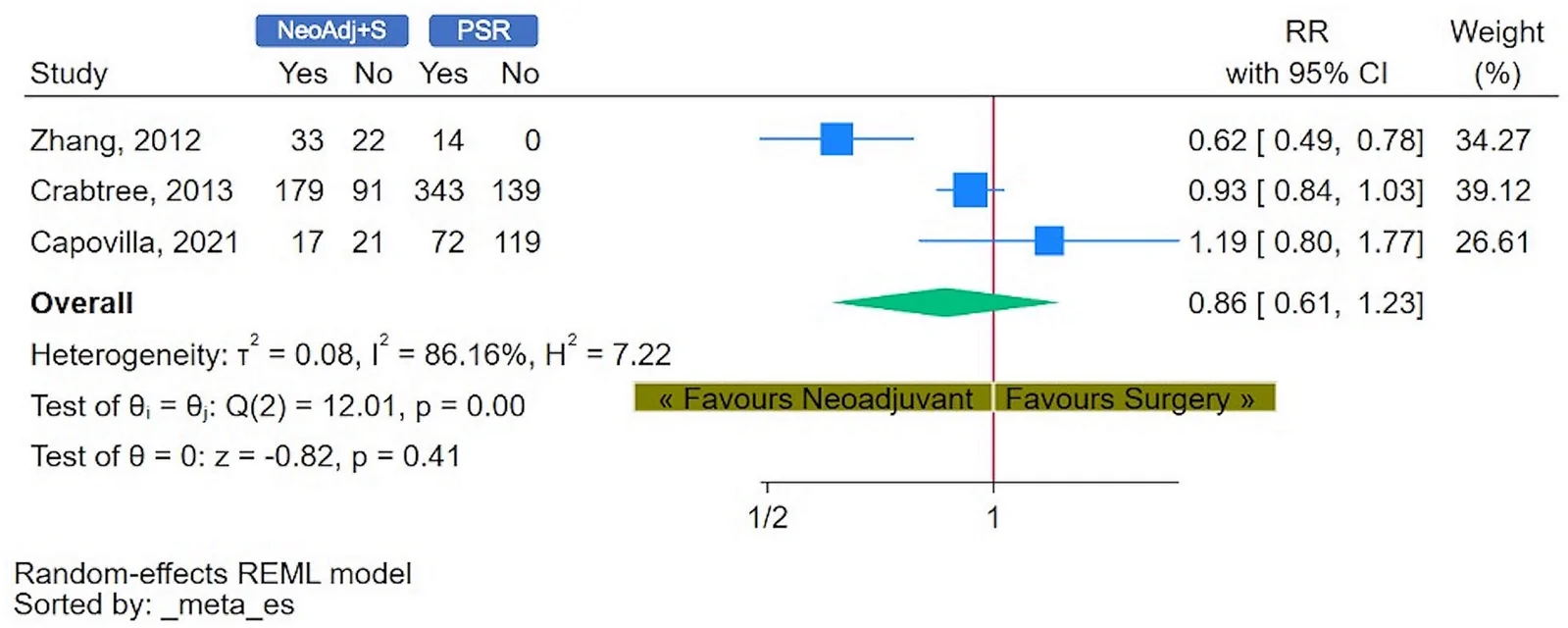
Forest plot of medical complications between Neoadjuvant therapy followed by surgery (NeoAdj + S) and Primary surgical resection (PSR). RR, risk ratios; IV, inverse variance; df, degrees of freedom.

Across medical complications, pulmonary complication was associated with a non-significant 12% decrease in NeoAdj+S compared to PSR (RR 0.88, 95% CI 0.77–1.01; I^2^ = 0%; low heterogeneity, favors NeoAdj+S) (Fig. 9A supplementary). NeoAdj+S was associated with a non-significant 23% increase in cardiovascular complication compared to PSR (RR 1.23, 95% CI 0.94–1.60; I^2^ = 0%; low heterogeneity, favors PSR) (Fig. 8B supplementary). NeoAdj+S was associated with a non-significant 32% decrease in infections compared to PSR (RR 0.68, 95% CI 0.28–1.69; I^2^ = 71.3%; substantial heterogeneity, favors NeoAdj+S) (Fig. 9C supplementary). NeoAdj+S was associated with a non-significant 24% decrease in other medical complications compared to PSR (RR 0.76, 95% CI 0.36–1.61; favors NeoAdj+S) (Fig. 9D supplementary).

### Sensitivity analysis

We performed sensitivity analyses using a leave-one-out approach to assess robustness of findings. No statistically significant results were achieved after this analysis.

### Risk of bias

All included studies demonstrated a high risk of bias. Only five (28%) studies achieved low risk of bias. The weakest domain was Comparability, with seven studies scoring poorly due to inadequate adjustment for key prognostic confounders (Table 3).

**Table 3:** Newcastle-Ottawa Scale (NOS) risk of bias assessment

**Study ID**	**Design**	**Selection (4 items, 4 points)**	**Comparability (1 items, 2 points)**	**Outcome (3 items, 3 points)**	**Total Risk of Bias score**
Capovilla 2021	Retrospective cohort study	★ ★ ★ ★	0	★	5
**Chen 2012**	Retrospective cohort study	★ ★ ★ ★	★ ★	★ ★	8
Crabtree 2013	Retrospective cohort study	★ ★ ★ ★	★	★	6
Dolan 2016	Retrospective cohort study	★ ★ ★	★	★ ★	6
**Goense 2018**	Retrospective cohort study	★ ★ ★ ★	★ ★	★ ★	8
Hardacker 2014	Prospective cohort study	★ ★ ★	★	★ ★	6
**Lin 2024**	Retrospective cohort study- Propensity matched	★ ★ ★ ★	★ ★	★ ★	8
Malin 2009	Prospective cohort study	★ ★ ★	0	★ ★ ★	6
**Markar 2016**	Retrospective cohort study	★ ★ ★ ★	★ ★	★ ★	8
**Martin 2013**	Retrospective cohort study	★ ★ ★ ★	★	★ ★	7
PerezHolguin 2021	Retrospective cohort study	★ ★ ★ ★	0	★ ★	6
Rhodin 2021	Retrospective cohort study	★ ★ ★ ★	0	★ ★	6
Rodriguez-Quintero 2024	Retrospective cohort study - Propensity matched	★ ★ ★	★	★ ★	6
Samson 2016	Retrospective cohort study	★ ★ ★ ★	★	★	6
Song 2016	Retrospective cohort study	★ ★ ★ ★	0	★ ★	6
Speicher 2014	Retrospective cohort study	★ ★ ★ ★	0	★ ★	6
Wirsik 2024	Retrospective cohort study	★ ★ ★ ★	0	★ ★	6
Zhang 2012	Retrospective cohort study	★ ★ ★	★	★ ★	6

**Bolded**: lowest risk of bias.

## DISCUSSION

Our systematic review and meta-analysis yielded 18 studies that represented 14,566 patients with a diagnosis of clinical T2N0 esophageal cancer of either SCC or EAC subtype. Of these, 7936 patients underwent neoadjuvant treatment in addition to resection, and 6630 patients underwent primary resection. No randomized controlled trials were identified; all included studies were cohort design, with patient data sourced from either a single-center or national databases. The majority (16 out of 18) were retrospective in nature.^9–24^ Overall, our meta-analysis did not find any statistically significant evidence favoring the use of either neoadjuvant therapy or primary surgical resection with respect to survival outcomes, R0 resection rates, or undesirable outcomes such as 30-day mortality and overall complication rates. For some outcomes, subgroup analysis, when stratified by neoadjuvant treatment regimens (chemotherapy only, radiotherapy only or chemoradiotherapy) or study design, did yield statistically significant results. However, these results were often inconsistent with the direction of the overall analysis (Central illustration).

Compared with prior systematic reviews published in 2018 and 2019, our review included similar studies but extends the existing literature in several important ways. Notably, this is the first systematic review to incorporate meta-regression analyses to examine potential moderators (mean age, male sex, and study sample size) and conduct subgroups analyses across multiple clinically relevant subgroups in patients with cT2N0 esophageal cancer. The 2018 review by Mota assessed outcomes similar to our review, such as 5-year survival, perioperative mortality, recurrence, and resection margins, and anastomotic leak complications, whereas Lv restricted their analysis to 5-year survival alone.^32^^,^^33^ In contrast, the 2019 systematic review and meta-analysis by Kidane focused primarily on clinical staging accuracy rather than comparative perioperative outcomes.^34^

Survival was the most commonly reported outcome among all included studies. In particular, 5-year survival was reported in 11 out of the 18 studies.^16–20^^,^^23–25^^,^^27^^,^^29^^,^^31^ Among all survival outcomes in our meta-analysis, there was no statistically significant difference between cohorts receiving neoadjuvant treatment compared to upfront resection. Of note, differing from previous meta-analyses, our study also did not demonstrate a statistically significant difference between R0 resection rates in the neoadjuvant treatment and upfront resection cohorts.^34^ It might be reasonable to surmise that this suggests neoadjuvant treatment did not downsize tumor burden sufficiently to result in less adjuvant treatment or improved survival in the T2N0 cohort. The absence of survival benefit and additional R0 resections therefore raises the question as to whether routine neoadjuvant treatment in this cohort results in any measurable benefit to patients. Although it is notable that we also did not demonstrate a statistically significant difference in perioperative mortality or overall, surgical or medical complication rates in this meta-analysis.

This systematic review and meta-analysis has several limitations. Most of the included studies were retrospective in design, drawing data from either national databases or single-center cohorts over long periods of time. With this, there is potential for data to be captured incompletely, or capturing data that is non-representative of the global population of patients with T2N0 esophageal cancer. Additionally, most included studies collated data over an average of 10 years despite recognizing that over this period, standard of treatment for esophageal cancer changed. Furthermore, we are unable to detect the impact of selection bias. The decision to offer neoadjuvant treatment or upfront surgery to participants is impacted by a multitude of individual factors in clinical settings including perceived fitness to undergo treatment due to co-morbidities or functional status, disease factors such as tumor location, or institutional variation. Among studies included in our review, it is not possible to assess the impact of this variability on resultant complications or survival outcomes. Among studies included in this review, one third employed propensity score matching or adjustment to address selection bias^21^^,^^25–27^^,^^29^ Future research might employ similar methods, inverse probability weighting or a randomized controlled trial design to reduce the effect of selection bias. We also restricted articles to English-language publications, which may introduce language bias. This is particularly relevant given that high-quality data on esophageal squamous cell carcinoma may be available from East Asian countries. Our review captures heterogenous data, both in regard to treatment regimen and tumor histology. Due to lack of outcome data by histological subgroups, we did not perform subgroup analyses comparing adenocarcinoma and squamous cell carcinoma, acknowledging their differing characteristics with regard to treatment response. This may obscure clinically important differences between histological subtypes and should be considered when interpreting the findings. Furthermore, patients were not consistently stratified by histopathological features such as lymphovascular invasion or tumor length.

Modality of clinical staging is an additional factor that was not controlled for among studies included in our systematic review and meta-analysis. Among studies that reported staging modality, multimodal investigations were utilized including computed tomography (CT), endoscopic ultrasound (EUS), positron emission tomography (PET), barium swallow or bone scan.^15–17^^,^^24–28^^,^^31^ Poor concordance between clinical and pathological staging has been previously described in this T2N0 cohort^7^ and is largely attributed to the limitations of currently available staging modalities. For locoregional disease, CT alone has poor diagnostic ability to detect tumor depth and nodal metastases and PET has a sensitivity of only 0.57 owing to the masking of nearby nodes by the signal from the primary tumor.^35^ EUS is noted to be limited by its ability to accurately detect the layers of the esophageal wall, and inter-operator accuracy as influenced by individual and institutional experience.^7^^,^^36^

Accuracy of clinical staging, although not the focus of this systematic review, was noted to be very heterogenous among the included studies. Most significantly, in one study 63.4% of patients were upstaged from their original T2N0 diagnosis.^31^ Wolfson’s systematic review and meta-analysis describes 29% accuracy of T staging.^7^ Upstaging of nodal status, was reported to occur in between 7% and 48.1% of cases among studies included in our analysis.^14–16^^,^^19^^,^^21–26^^,^^28^^,^^31^ This is of concern as selection of treatment modality on the basis of understaged disease undoubtedly increases risk of treatment failure. The likelihood of poorly concordant staging should be taken into consideration, therefore, for T2N0 patients. Particularly for patients with high-risk histological features such as a high-grade, poorly differentiated malignancy or evidence of lymphovascular invasion it may be reasonable to consider the use of neoadjuvant therapy.^37^

## CONCLUSION

The management of clinical T2N0 esophageal cancer remains a topical and challenging treatment dilemma. Our systematic review and meta-analysis revealed that evidence available in the literature, evaluating the use of neoadjuvant treatment, in this cohort is limited, and does not demonstrate a survival advantage nor increased morbidity or mortality with the use of neoadjuvant therapy. Inaccurate clinical staging is a major confounding factor in these findings. Further research incorporating standardized staging protocol and prospective designs is essential to establish unified, evidence-based recommendations for the management of clinical T2N0 esophageal cancer.

## Supplementary Material

Supplementary_material_doag113

## Notes

Funding statement: None.

Ethics approval statement: Not applicable.

Patient consent statement: Not applicable.
