# Supplementary material for: Management of cT2N0M0 esophageal cancer: a systematic review and meta-analysis
Source: Dis Esophagus. 2026 Oct 20;39(5):doag113. doi: 10.1093/dote/doag113 (PMC13682583; doi:10.1093/dote/doag113)
Supplement: Supplementary file 1 [file supplementary_material_doag113.docx]

**Supplementary appendix**

**Table 1.** Search strategy.

| **MEDLINE**  From 1946 to 25 November 2024 | | **EMBASE**  From 1947 to 25 November 2024 | | **CENTRAL**  From 1946 to 25 November 2024 | | **ClinicalTrials.gov**  On 25 November 2024 | |
| --- | --- | --- | --- | --- | --- | --- | --- |
| # |  | # |  | # |  | # |  |
| 1 | exp esophageal neoplasms/ (62262) | 1 | exp esophagus tumor/ (117103) | 1 | exp esophageal neoplasms/ (2674) | 1 | Esophageal T2N0 carcinoma |
| 2 | (oesophag* adj5 (neoplas* or cancer* or carcin* or tumo* or malig*)).tw. (9779) | 2 | (oesophag* adj5 (neoplas* or cancer* or carcin* or tumo* or malig*)).tw. (16305) | 2 | (oesophag* adj5 (neoplas* or cancer* or carcin* or tumo* or malig*)).tw. (1147) | 2 | Osophageal T2N0 carcinoma |
| 3 | (esophag* adj5 (neoplas* or cancer* or carcin* or tumo* or malig*)).tw. (58495) | 3 | (esophag* adj5 (neoplas* or cancer* or carcin* or tumo* or malig*)).tw. (84928) | 3 | (esophag* adj5 (neoplas* or cancer* or carcin* or tumo* or malig*)).tw. (5243) | 3 | Esophageal cancer |
| 4 | (adenocarcinoma$ adj5 esophag$).tw. (8894) | 4 | (adenocarcinoma$ adj5 esophag$).tw. (14693) | 4 | (adenocarcinoma$ adj5 esophag$).tw. (772) | 4 | Esophagogastric junction |
| 5 | Esophag* squamous cell carcinoma$.mp. (14910) | 5 | Esophag* squamous cell carcinoma$.mp. (26493) | 5 | Esophag* squamous cell carcinoma$.mp. (1151) |  |  |
| 6 | exp esophagogastric junction/ (10802) | 6 | or/1-5 (139931) | 6 | exp esophagogastric junction/ (727) |  |  |
| 7 | or/1-6 (93029) | 7 | "T2N0".ti,ab. (1331) | 7 | or/1-6 (7612) |  |  |
| 8 | "T2N0".ti,ab. (742) | 8 | "T2 N0".mp. (1021) | 8 | "T2N0".ti,ab. (98) |  |  |
| 9 | "T2 N0".mp. (616) | 9 | "T2 Stage".mp. (978) | 9 | "T2 N0".mp. (114) |  |  |
| 10 | "T2 Stage".mp. (589) | 10 | t2 n0 m0.mp. (318) | 10 | "T2 Stage".mp. (24) |  |  |
| 11 | t2 n0 m0.mp. (198) | 11 | ct2 n0 m0.mp. (15) | 11 | t2 n0 m0.mp. (25) |  |  |
| 12 | ct2 n0 m0.mp. (10) | 12 | ct2n0.mp. (218) | 12 | ct2 n0 m0.mp. (2) |  |  |
| 13 | ct2n0.mp. (113) | 13 | ct2n0m0.mp. (266) | 13 | ct2n0.mp. (11) |  |  |
| 14 | ct2n0m0.mp. (160) | 14 | t2n0.mp. (1334) | 14 | ct2n0m0.mp. (6) |  |  |
| 15 | t2n0.mp. (744) | 15 | or/7-14 (3713) | 15 | t2n0.mp. (102) |  |  |
| 16 | or/8-15 (2170) | 16 | 6 and 15 (**246 studies**) | 16 | or/8-15 (254) |  |  |
| 17 | 7 and 16 (**138 studies**) |  | -- | 17 | 7 and 16 (**11 studies**) |  |  |

**Figure 1A. Forest plot of 5-Years Overall Survival (OS) between Neoadjuvant therapy followed by surgery (NeoAdj+S) and Primary surgical resection (PSR) by NeoAdj type.**

**Figure 1B. Forest plot of 5-Years Overall Survival (OS) between Neoadjuvant therapy followed by surgery (NeoAdj+S) and Primary surgical resection (PSR) by Chemoradiotherapy protocols.**

**Figure 1C. Forest plot of 5-Years Overall Survival (OS) between Neoadjuvant therapy followed by surgery (NeoAdj+S) and Primary surgical resection (PSR) by Chemotherapy protocols.**

**Figure 1D. Forest plot of 5-Years Overall Survival (OS) between Neoadjuvant therapy followed by surgery (NeoAdj+S) and Primary surgical resection (PSR) by study design.**

## **Fig 1E: Funnel Plot for 5-Years Overall Survival (OS)**

## Publication bias tests

P-value from Egger test for os_5y: .226

P-value from Begg test for os_5y: .755

**Fig 1E: Trim and Fill Funnel Plot for 5-Years Overall Survival (OS)**

## **Table 1F: Trim and Fill Analysis for 5-Years Overall Survival (OS)**

|  | ES | Lower CI | Upper CI |
| --- | --- | --- | --- |
| Observed | -0.020 | -0.072 | 0.032 |
| Observed + Computed | -0.020 | -0.072 | 0.032 |

## **Fig 1G: Leave-One-Out Sensitivity Forest Plot for 5-Years Overall Survival (OS)**

## **Table 1H: Meta-regression Analysis for Fig 4: Leave-One-Out Sensitivity Forest Plot for 5-Years Overall Survival (OS)**

| _meta_es | Coefficient | Std. err. | z | P>\|z\| | [95% conf. interval] | |
| --- | --- | --- | --- | --- | --- | --- |
| mean_diff_age | 0.068 | 0.061 | 1.120 | 0.263 | -0.051 | .1862964 |
| male_percentage_diff | 0.009 | 0.033 | 0.280 | 0.781 | -0.055 | .0735747 |
| ss_total | -0.000 | 0.000 | -1.260 | 0.209 | -0.001 | .0001277 |
| _cons | 0.456 | 0.304 | 1.500 | 0.133 | -0.139 | 1.051093 |

**Figure 2A. Forest plot of 3-Years Overall Survival (OS) between Neoadjuvant therapy followed by surgery (NeoAdj+S) and Primary surgical resection (PSR) by NeoAdj type.**

**Figure 2B. Forest plot of 3-Years Overall Survival (OS) between Neoadjuvant therapy followed by surgery (NeoAdj+S) and Primary surgical resection (PSR) by Chemotherapy protocol.**

**Figure 2C. Forest plot of 3-Years Overall Survival (OS) between Neoadjuvant therapy followed by surgery (NeoAdj+S) and Primary surgical resection (PSR) by Chemotherapy protocols.**

**Figure 2D. Forest plot of 3-Years Overall Survival (OS) between Neoadjuvant therapy followed by surgery (NeoAdj+S) and Primary surgical resection (PSR) by study design.**

## **Fig 2E: Funnel Plot for 3-Years Overall Survival (OS)**

## Publication bias tests

P-value from Egger test for os_3y: .645

P-value from Begg test for os_3y: 1

## **Fig 2G: Trim and Fill Funnel Plot for 3-Years Overall Survival (OS)**

## **Table 1: Trim and Fill Analysis for 3-Years Overall Survival (OS)**

|  | ES | Lower CI | Upper CI |
| --- | --- | --- | --- |
| Observed | -0.022 | -0.071 | 0.027 |
| Observed + Computed | -0.022 | -0.071 | 0.027 |

## **Fig 2H: Leave-One-Out Sensitivity Forest Plot for 3-Years Overall Survival (OS)**

## **Table: Meta-regression Analysis for 3-Years Overall Survival (OS)**

NA due to nonconvergence or small number of studies

**Figure 3A. Forest plot of 5-Years Disease-Free survival (DFS) between Neoadjuvant therapy followed by surgery (NeoAdj+S) and Primary surgical resection (PSR) by NeoAdj type.**

**Figure 3B. Forest plot of 5-Years Disease-Free survival (DFS) between Neoadjuvant therapy followed by surgery (NeoAdj+S) and Primary surgical resection (PSR) by chemoradiotherapy protocol.**

**Figure 3C. Forest plot of 5-Years Disease-Free survival (DFS) between Neoadjuvant therapy followed by surgery (NeoAdj+S) and Primary surgical resection (PSR) by chemotherapy protocol.**

**Figure 3D. Forest plot of 5-Years Disease-Free survival (DFS) between Neoadjuvant therapy followed by surgery (NeoAdj+S) and Primary surgical resection (PSR) by study design.**

## **Fig 3G: Funnel Plot for 5-Years Disease-Free survival (DFS)**

## Publication bias tests

P-value from Egger test for dfs_5y: .015

P-value from Begg test for dfs_5y: .308

**Fig 3H: Trim and Fill Funnel Plot for 5-Years Disease-Free survival (DFS)**

## **Table 1: Trim and Fill Analysis for 5-Years Disease-Free survival (DFS)**

|  | ES | Lower CI | Upper CI |
| --- | --- | --- | --- |
| Observed | -0.032 | -0.129 | 0.065 |
| Observed + Computed | -0.032 | -0.129 | 0.065 |

**Fig 3G: Leave-One-Out Sensitivity Forest Plot for 5-Years Disease-Free survival (DFS)**

## **Table 2: Meta-regression Analysis for 5-Years Disease-Free survival (DFS)**

NA due to nonconvergence or small number of studies

**Figure 4A. Forest plot of longest reported survival between Neoadjuvant therapy followed by surgery (NeoAdj+S) and Primary surgical resection (PSR) by NeoAdj type.**

**Figure 4B. Forest plot of longest reported survival between Neoadjuvant therapy followed by surgery (NeoAdj+S) and Primary surgical resection (PSR) by Chemoradiotherapy protocols.**

**Figure 4C. Forest plot of longest reported survival between Neoadjuvant therapy followed by surgery (NeoAdj+S) and Primary surgical resection (PSR) by Chemotherapy protocols.**

**Figure 4D. Forest plot of longest reported survival between Neoadjuvant therapy followed by surgery (NeoAdj+S) and Primary surgical resection (PSR) by study design.**

**Figure 5A. Forest plot of R0 resection rate between Neoadjuvant therapy followed by surgery (NeoAdj+S) and Primary surgical resection (PSR) by NeoAdj type.**

**Figure 5B. Forest plot of R0 resection rate between Neoadjuvant therapy followed by surgery (NeoAdj+S) and Primary surgical resection (PSR) by chemoradiotherapy protocol.**

**Figure 5C. Forest plot of R0 resection rate between Neoadjuvant therapy followed by surgery (NeoAdj+S) and Primary surgical resection (PSR) by chemotherapy protocol.**

**Figure 5D. Forest plot of R0 resection rate between Neoadjuvant therapy followed by surgery (NeoAdj+S) and Primary surgical resection (PSR) by study design.**

## **Fig 5E: Funnel Plot for R0 resection rate**

## Publication bias tests

P-value from Egger test for r0_resec: .742

P-value from Begg test for r0_resec: 1

**Fig 5H: Trim and Fill Funnel Plot for R0 resection rate**

## **Table: Trim and Fill Analysis for R0 resection rate**

|  | ES | Lower CI | Upper CI |
| --- | --- | --- | --- |
| Observed | 0.026 | -0.001 | 0.053 |
| Observed + Computed | 0.026 | -0.001 | 0.053 |

**Fig 5G: Leave-One-Out Sensitivity Forest Plot for R0 resection rate**

## **Table:Meta-regression Analysis for R0 resection rate**

| _meta_es | Coefficient | Std. err. | z | P>\|z\| | [95% conf. interval] | |
| --- | --- | --- | --- | --- | --- | --- |
| mean_diff_age | 0.010 | 0.040 | 0.250 | 0.806 | -0.069 | .0889123 |
| male_percentage_diff | -0.028 | 0.017 | -1.670 | 0.095 | -0.061 | .0048936 |
| ss_total | -0.000 | 0.001 | -0.190 | 0.848 | -0.001 | .0011459 |
| _cons | 0.177 | 0.313 | 0.570 | 0.572 | -0.436 | .7897847 |

**Figure 6A. Forest plot of 30-days perioperative mortality between Neoadjuvant therapy followed by surgery (NeoAdj+S) and Primary surgical resection (PSR) by NeoAdj type.**

**Figure 6B. Forest plot of 30-days perioperative mortality between Neoadjuvant therapy followed by surgery (NeoAdj+S) and Primary surgical resection (PSR) by chemoradiotherapy protocols.**

**Figure 6C. Forest plot of 30-days perioperative mortality between Neoadjuvant therapy followed by surgery (NeoAdj+S) and Primary surgical resection (PSR) by chemotherapy protocols.**

**Figure 6D. Forest plot of 30-days perioperative mortality between Neoadjuvant therapy followed by surgery (NeoAdj+S) and Primary surgical resection (PSR) by study design.**

## **Fig 6E: Funnel Plot for 30-days perioperative mortality**

## **Publication bias tests**

P-value from Egger test for mort_30d: .543

P-value from Begg test for mort_30d: .221

## **Fig 6E: Trim and Fill Funnel Plot for 30-days perioperative mortality**

## **Table: Trim and Fill Analysis for 30-days perioperative mortality**

|  | ES | Lower CI | Upper CI |
| --- | --- | --- | --- |
| Observed | 0.003 | -0.326 | 0.331 |
| Observed + Computed | 0.003 | -0.326 | 0.331 |

## **Fig 6E: Leave-One-Out Sensitivity Forest Plot for 30-days perioperative mortality**

## **Table: Meta-regression Analysis for mort_30d**

NA due to nonconvergence or small number of studies

**Figure 7A. Forest plot of overall complications between Neoadjuvant therapy followed by surgery (NeoAdj+S) and Primary surgical resection (PSR) by NeoAdj type.**

**Figure 7B. Forest plot of overall complications between Neoadjuvant therapy followed by surgery (NeoAdj+S) and Primary surgical resection (PSR) by chemoradiotherapy protocol.**

**Figure 7C. Forest plot of overall complications between Neoadjuvant therapy followed by surgery (NeoAdj+S) and Primary surgical resection (PSR) by chemotherapy protocol.**

**Figure 7D. Forest plot of overall complications between Neoadjuvant therapy followed by surgery (NeoAdj+S) and Primary surgical resection (PSR) by study design.**

## **Fig 7E: Funnel Plot for overall complications**

## Publication bias tests

P-value from Egger test for any_complications: .325

P-value from Begg test for any_complications: .462

## **Fig 7F: Trim and Fill Funnel Plot for overall complications**

## **Table : Trim and Fill Analysis for overall complications**

|  | ES | Lower CI | Upper CI |
| --- | --- | --- | --- |
| Observed | -0.031 | -0.143 | 0.081 |
| Observed + Computed | -0.031 | -0.143 | 0.081 |

## **Fig 7F: Leave-One-Out Sensitivity Forest Plot for overall complications**

## **Table: Meta-regression Analysis for overall complications**

NA due to nonconvergence or small number of studies

**Figure 8A. Forest plot of surgical complications (anastomotic leak) between Neoadjuvant therapy followed by surgery (NeoAdj+S) and Primary surgical resection (PSR).**

**Figure 8B. Forest plot of surgical complications (chylothorax) between Neoadjuvant therapy followed by surgery (NeoAdj+S) and Primary surgical resection (PSR).**

**Figure 8C. Forest plot of surgical complications (tubular necrosis) between Neoadjuvant therapy followed by surgery (NeoAdj+S) and Primary surgical resection (PSR) by NeoAdj type.**

**Figure 8D. Forest plot of surgical complications (laryngeal nerve injury) between Neoadjuvant therapy followed by surgery (NeoAdj+S) and Primary surgical resection (PSR).**

**Figure 9A. Forest plot of medical complications (pulmonary) between Neoadjuvant therapy followed by surgery (NeoAdj+S) and Primary surgical resection (PSR).**

**Figure 9B. Forest plot of medical complications (cardiovascular) between Neoadjuvant therapy followed by surgery (NeoAdj+S) and Primary surgical resection (PSR).**

**Figure 9C. Forest plot of medical complications (infections) between Neoadjuvant therapy followed by surgery (NeoAdj+S) and Primary surgical resection (PSR).**

**Figure 9D. Forest plot of medical complications (other) between Neoadjuvant therapy followed by surgery (NeoAdj+S) and Primary surgical resection (PSR).**
